# The vitamin D system is deregulated in pancreatic diseases

**DOI:** 10.1016/j.jsbmb.2014.07.011

**Published:** 2014-10

**Authors:** Doris Hummel, Abhishek Aggarwal, Katalin Borka, Erika Bajna, Enikö Kállay, Henrik Csaba Horváth

**Affiliations:** a Department of Pathophysiology and Allergy Research, Medical University of Vienna, Währinger Gürtel 18-20, Leitstelle 3Q, 1090 Vienna, Austria; b 2nd Department of Pathology, Semmelweis University, Üllői út 93, 1091 Budapest, Hungary; c University Clinic of Visceral Surgery and Medicine, Inselspital University Hospital, 3010 Bern, Switzerland

**Keywords:** VDR, vitamin D receptor, CYP24A1, 1,25-dihydroxyvitamin D_3_ 24-hydroxylase, CaSR, calcium-sensing receptor, 1,25(OH)_2_D_3_, 1,25-dihydroxyvitamin D_3_, CYP27B1, 25-hydroxyvitamin D_3_ 1α-hydroxylase, VDRE, vitamin D response elements, PDAC, pancreatic ductal adenocarcinoma, CP, chronic pancreatitis, Vitamin D, CYP24A1, VDR, CaSR, Chronic pancreatitis, Pancreatic ductal adenocarcinoma, Pancreatic cancer

## Abstract

The vitamin D system is deregulated during development and progression of several cancer types. Data on the expression of the vitamin D system in the diseased pancreas are missing. The aim of this study was to investigate the expression of the vitamin D receptor (*VDR*), 1,25-dihydroxyvitamin D_3_ 24-hydroxylase (*CYP24A1*), and the calcium-sensing receptor (*CaSR*), a vitamin D target gene, in the different regions of the pancreas in patients with chronic pancreatitis (*n* = 6) and pancreatic ductal adenocarcinomas (PDAC) (*n* = 17). We analyzed the expression of these genes at mRNA and protein level with quantitative real-time RT-PCR and immunostaining. mRNA expression of *CYP24A1* and *VDR* was significantly increased in tumors compared with the adjacent non-tumorous tissue (*p* < 0.01), while *CaSR* mRNA expression decreased. Both the VDR and the CaSR protein were highly expressed in the endocrine compared with the exocrine pancreas. In CP the CYP24A1 expression was highest in the endocrine pancreas, while in PDACs in the transformed ducts. In the PDAC patients CYP24A1 expression in the islets was significantly lower than in CP patients. Our data suggest that during ductal adenocarcinoma development the vitamin D system in the pancreas becomes deregulated on two levels: in the islets CYP24A1 expression decreases weakening the negative feedback regulation of the vitamin D-dependent insulin synthesis/secretion. In the transformed ducts CYP24A1 expression increases, impairing the antiproliferative effect of vitamin D in these cells.

## Highlights

• During PDAC development CYP24A1 levels are reduced in the endocrine islets.
• During malignant transformation pancreatic ducts accumulate CYP24A1 protein.
• CYP24A1 expression correlates with VDR in CP patients, but not in PDAC patients.
• CYP24A1 overexpressing tumors are highly proliferative.
• CaSR and VDR are co-expressed in the endocrine cells of the islets.

## 1 Introduction

Diseases of the pancreas represent a large burden both for patients and for the public health system. Pancreatic cancer has poor clinical outcome, the 5-year survival rate is less than 6% (http://www.pancreatic.org^2^). The management and follow-up of these patients are still a major challenge for health care providers. Despite of relatively low incidence, death due to pancreatic cancer ranks fourth among cancer-related deaths in the Western world due to the poor survival rate and rapid fatality after diagnosis [1].

Vitamin D is a pluripotent secosteroid hormone, which plays an important role in a variety of physiologic and pathologic processes of the human body. In addition to its classical calciotropic effects, a whole spectrum of biological activities of 1,25-dihydroxyvitamin D_3_ (1,25(OH)_2_D_3_), the active form of vitamin D, have been detected during the last two decades in different tissues, e.g., breast, colon, and pancreas [2], [3]. These tissues – although not involved in the systemic regulation of 1,25(OH)_2_D_3_ levels – are able to activate and degrade vitamin D. 25-Hydroxyvitamin D_3_ 1α-hydroxylase (CYP27B1) hydroxylates the precursor 25-hydroxyvitamin D_3_ (25(OH)D_3_) at position C-1α to form the hormonally active metabolite 1,25(OH)_2_D_3_ [4]. Both 25(OH)D_3_ and 1,25(OH)_2_D_3_ are able to bind to the vitamin D receptor (VDR), which acts as a transcription factor and binds to the vitamin D response elements (VDRE) in the promoter of vitamin D target genes [5]. One of the main target genes of vitamin D bound to the VDR is 1,25-dihydroxyvitamin-D_3_ 24-hydroxylase (*CYP24A1*). This enzyme catabolizes 25(OH)D_3_ as well as 1,25(OH)_2_D_3_, resulting in the formation of calcitroic acid or 1α,25-(OH)_2_D_3_-26,23-lactone [6]. CYP24A1 is often overexpressed in cancer [7]. The promoter of the calcium sensing receptor (*CaSR*) gene, a G-protein coupled receptor, harbors two VDREs suggesting that *CaSR* is a vitamin D target gene [8]. In the pancreas, the CaSR is involved in regulating insulin secretion [9].

Studies have shown that 1,25(OH)_2_D_3_ has the potential to modulate both the endocrine and immune system [2]. Moreover, it plays a crucial role in regulating cellular processes like cell proliferation, differentiation, and apoptosis in an autocrine and/or paracrine manner, therefore, it is not surprising that the vitamin D system is deregulated in many forms of cancer [7].

The association between single nucleotide polymorphisms in genes of the vitamin D system and the risk of colorectal, breast, and prostate cancer has been described previously [10]. In a very recent study, Anderson et al. were able to link single nucleotide polymorphisms in vitamin D system genes and *CaSR* to pancreatic cancer risk [11].

While Mohr et al. found an inverse association between UV-B radiation which increases vitamin D production in the skin (the main source of vitamin D) and pancreatic cancer mortality [12], the results of studies trying to establish a link between dietary vitamin D intake and risk of pancreatic cancer are inconclusive. Skinner et al. [13] reported a reduction of the relative risk of pancreatic cancer with high vitamin D intake (≥600 IU/day), while a recent population-based study observed increased risk of pancreatic cancer in men with higher dietary vitamin D intake [14]. The results of cohort studies evaluating the association of circulating 25(OH)D_3_ levels with pancreatic cancer risk are further inconsistent; two studies by Stolzenberg-Solomon et al. reported an elevated risk for pancreatic cancer among patients with high 25(OH)D_3_ levels [15], [16], whereas another study from the same group found no association [17]. A very recent meta-analysis was unable to show any correlation between dietary vitamin D or 25(OH)D_3_ levels and risk of pancreatic cancer [18]. On the other hand, Wolpin et al. [19] showed that serum 25(OH)D_3_ levels below 45.6 nmol/L were associated with higher pancreatic cancer risk. Similarly, Bao et al. [20] found positive correlation between high predicted 25(OH)D_3_ score (predictors based on race, geographic region, vitamin D intake, body mass index, and physical activity) and lower risk for development of pancreatic cancer. A retrospective study showed that vitamin D deficiency and insufficiency were common among patients with adenocarcinoma of the pancreas and that there was a significant association between low vitamin D levels and poor outcome in patients with stage III and IV tumors [21].

The conflicting data on the role of circulating 25(OH)D_3_ in pancreatic carcinogenesis emphasize the need to study the role of the vitamin D system in pancreatic malignancies more in depth. We hypothesized that the expression of the vitamin D system is deregulated during pancreatic tumorigenesis. Therefore, in the present study, we evaluated the local expression of key factors of the vitamin D system and the vitamin D target gene *CaSR* in samples from chronic pancreatitis and pancreatic ductal adenocarcinoma in the different cell types of the pancreas to get new insights into the possible role of vitamin D-related pathomechanisms. Our results suggest that during the development of ductal adenocarcinoma the vitamin D system becomes impaired both in the endocrine and exocrine pancreas, affecting the cross-talk between these two systems.

## 2 Materials and methods

### 2.1 Patients

This study was conducted according to the Declaration of Helsinki. Written informed consent was obtained from all patients. Permission from the Ethics Commission of the Semmelweis University Medical School was granted prior to initiation of the study (ethical approval number 6626-1/2012/EKU). All consenting patients during the recruitment period were included in the analysis. We were able to obtain fresh material only in a subset of PDAC patients (*n* = 11). After surgical resection, parts of pancreatic tissue were frozen in −80 °C for RNA extraction, or formalin-fixed and paraffin embedded. Fresh frozen material was used for qRT-PCR and formalin-fixed paraffin embedded tissue was used for immunohistochemical and immunofluorescence staining. Demographic and pathological characteristics of patients who underwent pancreatoduodenectomy are shown in Table 1, Table 2 . Malignant tumors were diagnosed as pancreatic ductal adenocarcinoma (PDAC) according to the World Health Organization (WHO) classification [22].

**Table 1:** Pathological characteristics of the patient cohort used for mRNA analysis.

	Pancreatic ductal adenocarcinoma
Number of patients	11
Gender (male/female)	6/5
Age (median, range)	60 (46–71)
Tumor grade (TI/II/III)	0/4/7

TNM classification	
Primary tumor size (1/2/3/4)	0/1/10/0
Lymph node infiltration (N)	10
Distant metastasis (M) (site)	0

**Table 2:** Pathological characteristics of the patient cohort used for protien analysis.

	Chronic pancreatitis	Pancreatic ductal adenocarcinoma
Number of patients	6	17
Gender (male/female)	4/2	6/11
Age (median, range)	67 (32–74)	59 (46–74)
Tumor grade (TI/II/III)		1/8/8

TNM classification		
Primary tumor size (1/2/3/4)		0/3/12/2
Lymph node infiltration (N)		11
Distant metastasis (M) (site)		1 (liver)

### 2.2 RNA extraction, reverse transcription (RT), and quantitative real time RT-PCR

Total RNA from fresh frozen samples was isolated with TRIzol reagent (Invitrogen, Grand Island, NY, USA) according to the manufacturer's instructions. 2 μg of total RNA was reverse transcribed using RevertAid H Minus Reverse Transcriptase and Random Hexamer Primers (Fermentas, Ontario, Canada). Quantitative real time RT-PCR (qRT-PCR) was performed as described before [23]. We normalized relative expression of the target genes to the expression of two housekeeping genes suggested in the literature [24]: glutaminyl-tRNA synthetase (QARS), and polymerase (RNA) II (DNA directed) polypeptide (POL2RL). Expression levels were set relative to the calibrator (cDNA converted from total human RNA obtained from Clontech, Mountain View, CA, USA) and calculated according to the ΔΔC_T_ method. Primer sequences used were as follows: *QARS* (Fwd: GAGCGACTATTCCAGCAC, Rev: ATGCCAGGTTCAGGTCAC), *POL2RL* (Fwd: ACCGAGGGGGATGCGCTGGAT, Rev: CAGCGTGGTCACTTCTC, and *VDR* (Fwd: CGTCCAGCTTCTCCAATCTG, Rev: GTGAGGTCTCTGAATCCTGGT) were used. Primer sequences for *CYP24A1* [25], and the *CaSR* [26] have been published previously.

### 2.3 Immunohistochemistry

Immunohistochemistry for CYP24A1, VDR, and Ki67 was performed with a two-step indirect immunoperoxidase technique on 5 μm sections of formalin fixed paraffin-embedded tissue samples as described before [25]; VDR (dilution 1:100; Sigma–Aldrich, St. Louis, USA), Ki67 (dilution 1:100; Dako, Glostrup, Denmark). For immunohistochemical visualization of CaSR, samples were deparaffinized with xylol, rehydrated in ethanol and washed in phosphate buffered saline (PBS, pH 7.2), followed by antigen retrieval in EB-DEPP-9 buffer (1:20 in deionized water, Eubio, Vienna, Austria) for 10 min. Sections were then washed in PBS before permeabilization with 0.2% Tween-20 for 15 minutes and blocked in 5% fetal bovine serum in PBS. The samples were incubated with the primary antibody (monoclonal anti-mouse CaSR, 1:300, Abcam, Cambridge, UK) for one hour at room temperature. After a series of wash steps in PBS, staining was detected using the dextran polymer peroxidase Envision system (Dako, Glostrup, Denmark). The sections were washed, counterstained with Hematoxylin (Dako) and mounted as described before [25]. Normal kidney sections were used as positive controls. As negative control, the primary antibody was replaced with an isotype specific IgG.

### 2.4 Immunofluorescence staining

5 μm slides of formalin fixed paraffin embedded tissue (adenocarcinoma and chronic pancreatitis) were deparaffinized with xylol and rehydrated in ethanol. Antigen retrieval was performed in 0.05% citrate buffer, followed by wash steps in PBS. Samples were then permeabilized in 0.2% Tween, blocked in 5% goat serum and co-incubated with VDR antibody (1:100, Sigma–Aldrich) and synaptophysin antibody (1:100, Biogenex, Fremont, CA, USA) or VDR antibody and CaSR antibody (1:200, Abcam). After washing, samples were incubated with Dylight 549 goat-anti-rabbit IgG (1:500, Vector Laboratories, Burlingame, CA, USA) and Alexafluor 647 goat-anti-mouse IgG (1:1000, Jackson ImmunoResearch, Suffolk, UK). Nuclei were stained with 4′,6-diamidino-2-phenylindole (DAPI, Roche, Basel, Switzerland) and mounted with Fluoromount-G (Southern Biotech, Birmingham, AL, USA).

### 2.5 Automated analysis of stained sections

Images of whole tissue sections were acquired using TissueFAXS (TissueGnostics GmbH, Vienna, Austria) [27]. We analyzed the number of positive cells using the HistoQuest software (TissueGnostics GmbH). This software allows automated acquisition of both immunofluorescence and immunohistochemistry images. After selection of the regions of interest (acinar, ductal, stromal, endocrine, and tumor cells) by a pathologist, quantitative analysis of the percentage of positive staining was carried out using HistoQuest. We analyzed 5000 cells per slide for each cell type (acinar, endocrine, stromal, tumor and ductal). The mean area of each investigated region was 3.4 mm^2^. Fluorophores used for immunofluorescence were: DAPI (for nucleus), TxRed (for VDR), and Cy5 (for CaSR and synaptophysin). Using extended focus, a feature of this software, we were able to ensure correct focus by merging several z-stacks into one sharp image. Images were acquired at exposure times where IgG controls were completely negative to ensure that unspecific staining was gated out.

### 2.6 Statistical analysis

We used SPSS statistics package, v18.0 for statistical analysis and GraphPadPrism v5.0 for designing graphs. We performed paired *t*-tests on log2-transformed, normally distributed data for mRNA expression. An outliers test was performed using GraphPad QuickCalcs and outliers were excluded from analysis. We used Wilcoxon signed ranks test for analyzing protein expression in patients with CP or PDAC, and the Mann–Whitney *U*-test to compare between disease groups. These tests were corrected for multiple comparisons using a Bonferroni–Holm correction. *P*-values <0.05 were considered significant. Correlation analysis was performed by calculation of Spearman's correlation coefficient (*ρ*) with two-tailed significance levels.

## 3 Results

### 3.1 mRNA expression of VDR, CYP24A1, and CaSR in pancreatic ductal adenocarcinoma (PDAC)

**Fig. 1:**
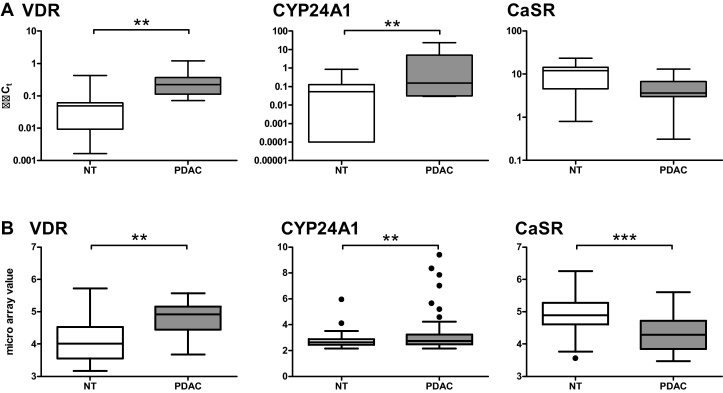
mRNA expression of the VDR and CYP24A1 is significantly increased and CaSR expression decreases in PDAC compared with adjacent non-tumorous (NT) parts of the pancreas. (A) mRNA expression (*n* = 11) was assessed by qRT-PCR and set relative to calibrator. (B) Data originate from a microarray analysis of mRNA from patients suffering from PDAC [28]. Median, interquartile range and whiskers according to Tukey are shown. Statistics were calculated using paired *t*-test on log2-transformed data. Among the values for CYP24A1 expression we identified one outlier in each dataset. (**p < 0.01, ***p < 0.001).

In our patient cohort (Table 1), we observed a significant upregulation of *VDR* (mean 3.7-fold) as well as of *CYP24A1* (mean 30.78-fold) mRNA expression in PDAC compared with adjacent parts of the pancreas. mRNA expression of the *CaSR* decreased in the tumors of most patients by 50%, however did not reach statistical significance (Fig. 1A). CYP27B1 levels did not change (data not shown). Our data are consistent with the microarray gene expression data published in the GEO database by Zhang et al. [28] (Fig. 1B), suggesting that our patient cohort was representative, despite the low sample size.

### 3.2 Protein expression and localization of the VDR in patients with chronic pancreatitis (CP) and pancreatic ductal adenocarcinoma (PDAC)

**Fig. 2:**
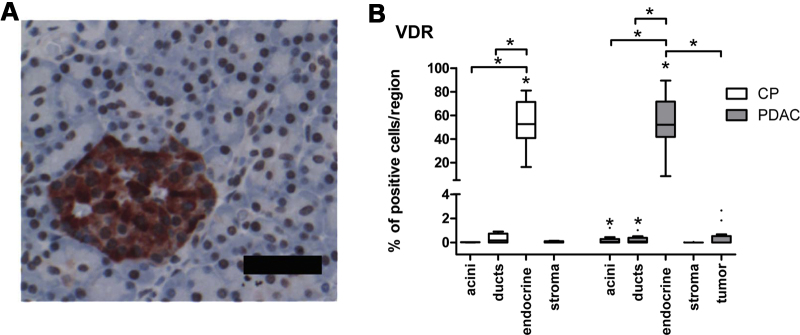
Protein expression of VDR in CP and PDAC. VDR is highly expressed in endocrine islets compared with other parts of the pancreas. (A) IHC of an endocrine islet in a region of chronic pancreatitis is shown. Black bar equals 50 μm. (B) Protein expression was determined using IHC, percentage of VDR positive cells is shown. Median, interquartile range and whiskers according to Tukey are shown. Wilcoxon signed ranks test was applied to compare protein expression in patients with CP and PDAC, and Mann–Whitney *U*-test to compare between disease groups. Both tests were followed by Bonferroni–Holm correction for multiple comparisons. Asterisks above boxes indicate statistically significant difference from stroma (*p < 0.05).

In our patient cohort (Table 2) we analyzed expression and localization of the VDR in different regions of the pancreatic tissue. Expression of VDR was significantly higher in the endocrine islets compared with all other regions of the pancreas in patients with CP as well as in patients with PDAC. In PDAC patients, VDR expression was significantly higher also in acinar and ductal cells compared with the stroma (Fig. 2).

### 3.3 Protein expression and localization of CYP24A1 in patients with chronic pancreatitis (CP) and pancreatic ductal adenocarcinoma (PDAC)

**Fig. 3:**
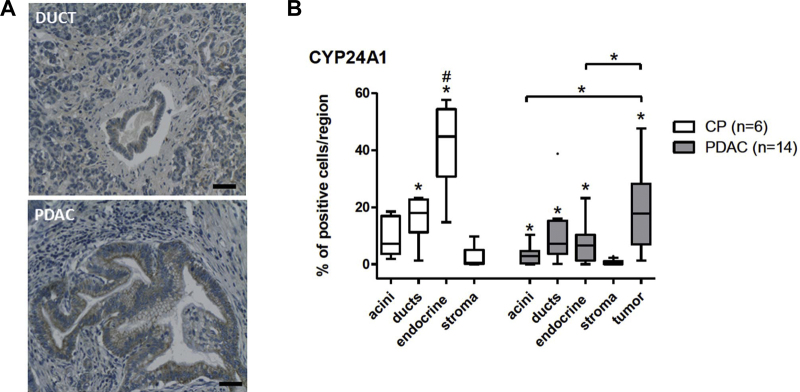
Expression of CYP24A1 in pancreatic ductal adenocarcinoma compared with non-tumorous regions of the same patient. (A) IHC of CYP24A1 in a patient with PDAC is shown. Black bar equals 50 μM. (B) Protein expression of the CYP24A1 is increased in ductal adenocarcinoma compared with other regions of pancreatic tissue. Protein expression was determined using IHC, percentage of CYP24A1 positive cells is shown. Median, interquartile range and whiskers according to Tukey are shown. Wilcoxon signed ranks test was applied to compare protein expression in patients with CP and PDAC, and Mann–Whitney *U*-test to compare between disease groups. Both tests were followed by Bonferroni–Holm correction for multiple comparisons. Asterisks above boxes indicate statistically significant difference from stroma (*p < 0.05). Hashtags indicate statistically significant difference between patients with CP and PDAC (#*p *< 0.05, ##*p *< 0.01). (For interpretation of the references to color in this figure legend, the reader is referred to the web version of this article.)

We analyzed expression of CYP24A1 in different regions of the pancreatic tissue (Fig. 3A). In CP patients, CYP24A1 expression varied significantly among the different regions of the pancreas, increasing in the following order: stroma < acinar cells < ducts < endocrine islets. In PDAC patients, the tumor expressed significantly more CYP24A1 than the acini, endocrine islets, or stroma (Fig. 3B). The endocrine islets of PDAC patients expressed significantly less CYP24A1 than the islets of CP patients (Fig. 3B).

CYP24A1 is a VDR target gene; therefore we tested whether CYP24A1 expression correlates with VDR expression. Spearman's correlation analysis showed that CYP24A1 and VDR correlated strongly in CP patients; however, this correlation was lost in PDAC patients (Table 3).

**Table 3:** VDR expression correlates with CYP24A1 expression in CP but not in PDAC patients.

		VDR	
		CP	PDAC
CYP24A1	Correlation coefficient	0.715	0.206
	*p*-value	<0.001	Not significant

### 3.4 Protein expression of Ki67 in patients with chronic pancreatitis (CP) and pancreatic ductal adenocarcinoma (PDAC)

**Fig. 4:**
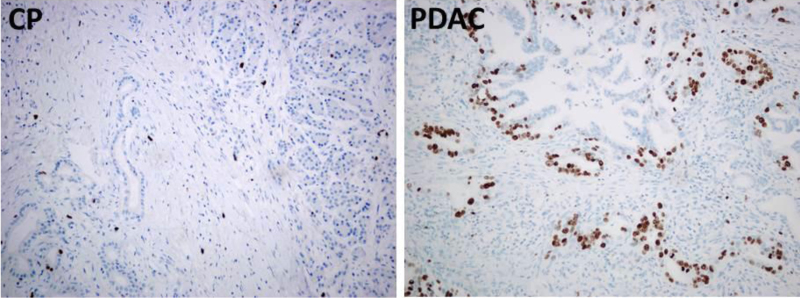
Expression of Ki67 in chronic pancreatitis and pancreatic ductal adenocarcinoma. CP patients have low expression of the proliferative marker Ki67. In PDAC patients Ki67 expression is increased in tumor compared with non-tumorous regions of the same patient. Immunostaining of Ki67 (brown) in patient with CP (left) and PDAC (right) is shown. (For interpretation of the references to color in this figure legend, the reader is referred to the web version of this article.)

We analyzed expression and intensity of the proliferation marker, Ki67 in different regions of the pancreatic tissue. In PDAC samples, Ki67 expression was much stronger in the tumor compared with the other regions of the tissue (Fig. 4). In CP patients Ki67 staining was very weak in all regions analyzed. Spearman's correlation analysis showed significant correlation between CYP24A1 and Ki67 in the tumor and stroma of the PDAC patients. In CP patients CYP24A1 and Ki67 expression did not correlate (Table 4).

**Table 4:** Ki67 expression correlates with CYP24A1 expression in PDAC but not in CP patients.

		Ki67	
		CP	PDAC
CYP24A1	Correlation coefficient	−0.377	0.409
	*p*-value	Not significant	<0.01

### 3.5 Protein expression and localization of the calcium-sensing receptor (CaSR) in patients with (CP) and pancreatic ductal adenocarcinoma (PDAC)

**Fig. 5:**
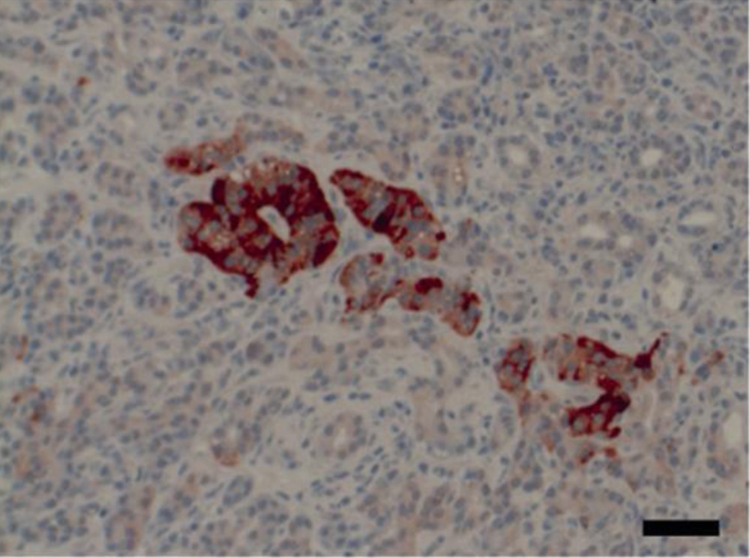
CaSR expression is high in endocrine islets compared with other parts of the pancreas. IHC of an endocrine islet with surrounding acinar and duct cells is shown. Black bar equals 50 μm.

Expression of the vitamin D target gene, CaSR, was strongest in the endocrine islets in all samples. CaSR protein was expressed also in the acinar and duct cells, however at much lower level (Fig. 5). Staining intensity of ducts and acini was comparable between non-tumorous and cancerous tissue, consistent with previous findings [29].

### 3.6 Co-expression of VDR and CaSR protein in endocrine cells

**Fig. 6:**
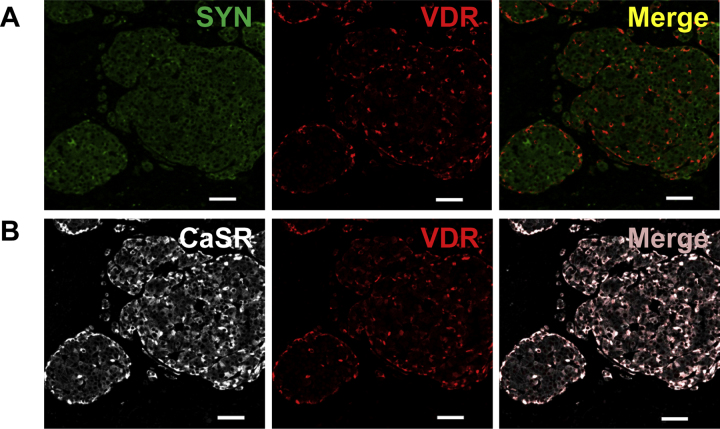
VDR and the CaSR are expressed in the endocrine cells of the pancreas. (A) Immunofluorescence staining of the endocrine marker synaptophysin (SYN, green) and the VDR (red) and the merged picture. (B) Immunofluorescence staining of the CaSR (white) and the VDR (red) and the merged picture. White bar equals 50 μM.

In order to assess whether the VDR and CaSR were expressed in the same cells of the endocrine islets, we performed dual immunofluorescence staining. We stained VDR/synaptophysin and CaSR/VDR in combination and were able to show that VDR co-localizes with synaptophysin (Fig. 6A) and is expressed in the same cells as the CaSR (Fig. 6B), implying a co-expression of CaSR with synaptophysin. Thus, we show that the cells expressing the VDR and the CaSR are the synaptophysin expressing endocrine cells of the islets.

## 4 Discussion

In this study, we investigated the expression of VDR, CYP24A1 and the vitamin D target gene CaSR in diseased pancreatic tissue. We show for the first time that the expression of the vitamin D degrading enzyme, CYP24A1 is increased both during inflammation (as in chronic pancreatitis) and during malignant transformation (as in pancreatic ductal adenocarcinoma). In all regions of the pancreas CYP24A1 expression is generally higher in CP patients than in PDAC patients. By comparing expression of the studied proteins in the various cell types of the pancreas, we are the first to show that during ductal adenocarcinoma development, the islet cells lose CYP24A1 expression while the transformed ductal cells up-regulate CYP24A1 expression.

The pancreas is a very complex organ, consisting of an endocrine and exocrine part. Therefore, we examined the expression pattern of the two of most-relevant genes for vitamin D metabolism and signaling (VDR and CYP24A1) and also included one vitamin D target gene (*CaSR*) in the different morphological structures of the pancreas, such as acinar cells, ducts, endocrine cells, and stroma as well as tumor regions from patients with PDAC. mRNA levels of the vitamin D activating enzyme, 25-hydroxyvitamin D3-1-alpha hydroxylase (CYP27B1) was unchanged in our patient cohort (data not shown).

In CP patients, we found highest CYP24A1 levels in the endocrine cells. Interestingly, in PDAC patients, the tumor cells expressed more CYP24A1 than any other cell type, even more than the endocrine cells. It seems that during malignant transformation, the untransformed endocrine cells lose their CYP24A1 expression, while the transformed ductal cells accumulate CYP24A1. Whether this is causally linked with the transformation process or it is a consequence of this process, needs to be determined.

The vitamin D degrading enzyme *CYP24A1* is the main target gene of the hormonally active form of vitamin D, 1,25(OH)_2_D_3_ and is most abundant in the kidney, but it is also expressed in several other tissues [30], [31], [32], [33]. The expression of CYP24A1 is increased in several malignancies, such as colon, ovary, breast, lung, and esophageal tumors [34], [35], [36], [37]. This overexpression probably leads to an immediate degradation of the locally available 1,25(OH)_2_D_3_, impairing its antitumorigenic action in the tumor tissue. *CYP24A1* gene transcription is initiated upon binding of the VDR/1,25(OH)_2_D_3_-complex to the VDREs in the gene promoter. The simultaneous up-regulation of *CYP24A1* and *VDR* mRNA in PDAC could lead to the assumption of a VDR-dependent *CYP24A1* mRNA enhancement. This seems to be the case in CP patients, where CYP24A1 expression correlates with VDR significantly. However, this correlation is lost in PDAC patients where VDR protein levels are highest in the endocrine cells while the CYP24A1 is highest in tumor cells.

From these data we speculate that up-regulation of CYP24A1 in the tumor is uncoupled from VDR, and therefore is independent of the local levels of 1,25(OH)_2_D_3_, as it has been suggested for benign and malignant tumors of the colon [25]. The high CYP24A1 levels would degrade the locally available 1,25(OH)_2_D_3_, preventing its antitumorigenic action. One reason leading to CYP24A1 overexpression in pancreatic tumors could be an activating mutation of K-ras. K-ras mutations occur in almost all advanced PDAC cases [38], [39]. Zhang et al. have shown that lung cancer cell lines harboring a K-ras mutation express high CYP24A1 levels, while VDR expression is low [40]. Anderson et al. have shown recently that single nucleotide polymorphisms in *CYP24A1* correlate with risk of pancreatic cancer [11]. Whether this particular modification has any effect on the activity or expression of CYP24A1 is not known.

We found the highest expression of VDR and CaSR in the endocrine cells of the pancreas while the exocrine pancreas expressed much lower levels. The up-regulation of VDR in pancreatic cancer cells compared with normal cells has been shown *in vitro* in primary tumor cells, supporting our present results on mRNA level [41]. Pancreatic cancer cells responded to treatment with an 1,25(OH)_2_D_3_ analogue by inhibition of proliferation [42], [43]. Whether up-regulation of the VDR in pancreatic cancer can be seen as a defense mechanism to make use of the locally available 1,25(OH)_2_D_3_ more efficiently remains to be investigated. Interestingly, single nucleotide polymorphisms in the *VDR* gene are associated with a higher risk for pancreatic cancer [11], [44]. These results emphasize the possible impact of the vitamin D receptor in malignant transformation of the pancreas.

In our samples, VDR protein is expressed much higher in the endocrine islets compared with all other regions in CP as well as in PDAC samples. These results suggest that VDR, and consequently vitamin D, play a pivotal role in the endocrine function of the pancreas; however, it might be less important in exocrine secretion.

It has been shown previously that mice lacking the VDR have impaired insulin secretion [45]. The role of vitamin D and involvement of the VDR in the pathogenesis of insulin-dependent diabetes mellitus (diabetes type 1) has been studied extensively [46]. A link between type 1 diabetes and VDR in the β-cells of the endocrine islets has been suggested previously [47], [48]. Wolden-Kirk et al. showed recently that 1,25(OH)_2_D_3_ protects β-cells from cytokine induced apoptosis and impaired insulin secretion [49]. Moreover, the promoter of the human insulin gene harbors a VDRE [50]. It seems that 1,25(OH)_2_D_3_ increases both synthesis and secretion of insulin [51] therefore 1,25(OH)_2_D_3_ levels in the endocrine cells are tightly regulated by CYP24A1. At the same time, insulin promotes pancreatic cancer in both epidemiologic and animal studies either directly or indirectly [52], [53].

In the endocrine cells of PDAC patients, CYP24A1 expression was significantly lower than in the patients with CP (and probably in the normal pancreas), while it was increased in the tumor cells. We speculate that the tight regulation of 1,25(OH)_2_D_3_ levels, needed in these cells for a balanced vitamin D-dependent regulation of insulin secretion is lost. As a consequence, higher vitamin D levels in patients with low CYP24A1 levels in the endocrine cells would lead to enhanced insulin secretion. This could then promote growth of neighboring tumor cells in a paracrine manner. Additionally, in the tumor itself, the high CYP24A1 levels would catabolize 1,25(OH)_2_D_3_ immediately, impairing its anti-tumorigenic effects. Indeed, in PDAC patients CYP24A1 levels correlated significantly with Ki67 expression, suggesting that tumors overexpressing CYP24A1 are highly proliferative. This hypothesis might explain the positive associations observed in some studies between vitamin D and pancreatic cancer, however it needs further proof.

It seems that vitamin D and calcium could co-regulate production of insulin. Faure-Dussert et al., in a study using vitamin D-deficient rat islets, demonstrated increased β-cell sensitivity to 1,25(OH)_2_D_3_-dependent insulin secretion under hypocalcemic conditions [54]. Furthermore, Squires et al. have shown that the CaSR negatively modulates secretion of insulin in the pancreas [9]. In our patients we show co-expression of VDR and CaSR in the endocrine islets leading us to speculate a cross talk between these molecules in the pancreatic islets.

The main limitations of this descriptive pilot study are the small patient number and the lack of normal tissue. Its strength is that we examined the expression of the vitamin D system in the different cell types of the pancreas and found a cell type-dependent deregulation of the vitamin D pathway in ductal adenocarcinoma development.

## 5 Conclusions

In this study, we show for the first time that CYP24A1 is overexpressed in pancreatic tumors on mRNA as well as on protein level. In chronic pancreatitis patients the significant correlation of CYP24A1 and VDR suggests that high CYP24A1 expression in the islets is a physiological up-regulation of CYP24A1. This correlation is lost during malignant transformation. Further, our data suggest that during ductal adenocarcinoma development the vitamin D system in the pancreas becomes deregulated on two levels: In the islets CYP24A1 expression decreases weakening the negative feedback regulation of the vitamin D-dependent insulin synthesis and secretion. The higher insulin levels could promote tumor growth. In the transformed ducts CYP24A1 expression increases, impairing the antiproliferative effect of vitamin D in these cells.

## Conflict of interest

The authors have no conflicts of interest to declare.

## Funding

This study was funded by the Stiftung Aktion Österreich-Ungarn 82ÖU16, the Austrian Science Fund, Project #P22200-B11, and the EU Marie Curie ITN #264663.

## Footnotes

2 Accessed on 21st of August 2014.
